# Anther-derived microspore embryogenesis in pepper hybrids orobelle and Bomby

**DOI:** 10.1186/s40529-023-00408-6

**Published:** 2024-01-04

**Authors:** K. P. Sahana, Arpita Srivastava, Anil Khar, Neelu Jain, P. K. Jain, Hemlata Bharti, Mohd Harun, Manisha Mangal

**Affiliations:** 1 Division of Vegetable Science, ICAR-Indian Agricultural Research Institute, New Delhi, India; 2 Division of Genetics, ICAR-Indian Agricultural Research Institute, New Delhi, India; 3 ICAR - National Institute for Plant Biotechnology, New Delhi, India; 4 Centre for Protected Cultivation Technology, ICAR-Indian Agricultural Research Institute, New Delhi, India; 5 Division of Design and Experiments, ICAR-IASRI, Indian Agricultural Research Institute, New Delhi, India

**Keywords:** Anther culture, *Capsicum annuum*, Explant, Homozygous lines

## Abstract

**Background:**

Traditional breeding methods have long been employed worldwide for the evaluation and development of pepper cultivars. However, these methods necessitate multiple generations of screening, line development, evaluation, recognition, and crossing to obtain highly homozygous lines. In contrast, in vitro anther-derived microspore culture represents a rapid method to generate homozygous lines within a single generation. In the present study, we have optimized a protocol for microspore embryogenesis from anther cultures of pepper hybrids Orobelle and Bomby.

**Results:**

We achieved early and successful embryo formation from both genotypes by subjecting the buds to a cold pretreatment at 4 °C for 4 days. Our optimized culture medium, comprised of MS medium supplemented with 4 mg/L NAA, 1 mg/L BAP, 0.25% activated charcoal, 2.6 g/L gelrite, 30 g/L sucrose, and 15 mg/L silver nitrate, exhibited the highest efficiency in embryo formation (1.85% and 1.46%) for Orobelle and Bomby, respectively. Furthermore, successful plant regeneration from the anther derived microspore embryos was accomplished using half-strength MS medium fortified with 2% sucrose and 0.1 mg/L 6-benzylaminopurine (BA), solidified with 2.6 g/L gelrite. The ploidy status of the microspore-derived plantlets was analyzed using flow cytometry technique. Notably, the haploid plants exhibited distinct characteristics such as reduced plant height, leaf length, leaf width, and shorter internode length when compared to their diploid counterparts derived from seeds.

**Conclusion:**

Our findings highlight the potential of anther culture and microspore embryogenesis as an advanced method for accelerating pepper breeding programs, enabling the rapid production of superior homozygous lines.

## Introduction

Capsicum, commonly known as sweet pepper or bell pepper, holds immense economic importance as a crop within the Solanaceae family. Indigenous to tropical and subtropical America (Hunziker 2001), this plant is revered for its blocky, round, conical fruits with thick flesh and non-pungent taste. Across the globe, it is celebrated as a highly sought-after exotic vegetable, adorning numerous continental culinary preparations. All pepper populations are diploid and have the same chromosome number (2n = 2x = 24). The genome size of hot pepper is ~ 3.5 Gb, with 75 to 80% of the genome composed of repetitive elements (Saisupriya and Saidaiah 2021). The allure of Capsicum extends beyond its delightful flavor and fragrance; it boasts remarkable nutritional value, featuring high levels of provitamin A, vitamin C, and an array of essential minerals such as iron, potassium, calcium, magnesium, phosphorus, sodium, and selenium (Chassy et al. 2006; Agarwal et al. 2007). As a center of diversity for *C. annuum*, Mexico stands as a testament to the rich variability present within the genus Capsicum, which encompasses over 35 species, including the domesticated *C. annuum*, *C. frutescens*, *C. chinense*, *C.baccatum*, and *C. pubescens* (Carrizo et al. 2016).

With its cultivation spanning 126 countries worldwide, China ranks as world’s largest producer followed by the Mexico (FAOSTAT 2019). The World pepper area and production has been reported to be 1.99 mha and 38.02MT respectively (FAOSTAT 2019). However, despite its popularity, cultivated pepper varieties are susceptible to various biotic and abiotic stresses that adversely impact crop productivity. Thus, the primary breeding objective in sweet pepper entails the development of stress-resistant varieties. Traditional breeding methods often rely on purelines, necessitating labor-intensive and time-consuming processes involving six to seven generations of self-fertilization to obtain pure homozygous lines.

To expedite the breeding program and overcome the challenges inherent in conventional methods, biotechnological approaches utilizing in vitro tissue culture techniques have gained substantial attention. Anther and microspore culture techniques have emerged as invaluable tools for plant breeders and geneticists seeking to generate new genetic variability and produce doubled haploid lines. Particularly, the anther culture technique offers the remarkable advantage of obtaining complete homozygous inbred lines in a single generation, significantly reducing the number of selfings and lowering production costs (Seguí-Simarro 2010). Furthermore, microspore embryogenesis serves as a valuable culture technique for obtaining complete homozygous lines solely from the male gametes in pepper breeding (Comlekcioglu and Ellialtioglu 2018). The groundbreaking successes of Wang et al. (1973) in China and George and Narayanaswamy (1973) in India, who achieved the development of haploid embryos from *C. annuum* anthers, have set the stage for pioneering advancements in this field. Moreover, the initiation of in vitro androgenesis studies on domestic pepper genotypes by Abak (1983) in Turkey signifies the global significance and ongoing exploration of these techniques. 

Nevertheless, the intricate process of haploid embryo development and germination remains a significant challenge, impeding the efficient production of doubled-haploid peppers (Seguí Simarro 2016). The intricate interplay of various factors, including the genotype of the donor plant, the developmental stage of microspores, anther pretreatments, optimal media combinations, and precise in vitro culture conditions, all play decisive roles in achieving successful outcomes (Çiner and Tipirdamaz 2001; Nowaczyk and Kisiala 2006). The inherent recalcitrance of pepper species towards androgenesis further underscores the criticality of identifying responsive genotypes to achieve successful regeneration of haploid embryos (Irikova et al. 2011a, b).

In light of these challenges, our study embarked on the task of developing an efficient protocol for haploid development in the pepper hybrids, Orobelle and Bomby. Both are commercial F1 capsicum hybrids from Syngenta India Private Limited (Pune, Maharashtra, India). These hybrids were selected for the present study as they have consisitantly performed well and given maximum sustainable yield and quality under North Indian Plains. We investigated the effects of various pre- and post-culture treatments, the season of bud collection, as well as the type and components of nutrient media on the efficiency of anther derived microspore embryo formation. By systematically exploring these factors, our study aimed to enhance the understanding and optimization of haploid development protocols specifically tailored for these pepper hybrids. The findings of our study hold practical implications for the development of new pepper varieties with improved traits.

## Materials and methods

### Plant material

In the present study, two commercial hybrids of *Capsicum annuum* namely Orobelle and Bomby were used as the test genotypes. Seeds were sown in seedling trays containing coco peat: vermiculite: perlite in a ratio of 3:1:1 in the greenhouse during the month of October 2019 at Centre for Protected Cultivation Technology, ICAR-Indian Agricultural Research Institute, New Delhi, India. 42 days old seedlings were transplanted into polyhouse with controlled temperature conditions (16 h of light at 27 ± 2 °C and 8 h dark at 19 ± 2 °C, 925 µmol m^−2^ s^−1^ light intensity and 80% relative humidity) and raised as healthy donor plants. Adequate nutrition to the plants were provided by adding compost, well-rotten farm yard manure, and other organic matter to the soil. Soil moisture was maintained through regular irrigation using a drip system, while taking care to prevent waterlogging. Fertigation at three weeks intervals after transplanting using N:P:K 19:19:19 at a rate of 300 g in 100 L of water on a fortnightly basis was given to promote better plant growth and fertilizer doses were increased as the plants grew. We maintained optimal temperature and humidity levels inside the polyhouse to ensure healthy growth, with temperatures between 20^0^–30 °C during the day and 15–20 °C at night. To control pest populations, regular spraying with Difenthioron@0.5 g/ltr and Spiromesifen @0.5 ml/ltr was done. The plants were pruned regularly to remove any dead or diseased leaves, which could make the plants more vulnerable to pests and diseases. The seeds of donor genotypes were sown in staggered manner at fifteen days intervals to get continuous availability of buds for in vitro culture.

## Anther culture

### Selection of appropriate Buds for anther culture

Buds for experimentation were taken from the first flush (new growth of flowers that emerge at the same time) of flowering during morning hours. The collected buds were immediately transported to the laboratory in cool and moist condition to maintain their viability. In our previous study (Sahana et al. 2021) attempts were made to establish correlation between bud and anther parameters with stage of microsporogenesis/microgametogenesis so that appropriate microspore development stage can be identified by using DAPI staining method. For the current investigation, buds and anthers containing uninucleate pollens were chosen based on standardized morphological parameters established in that study. These parameters included selecting flower buds with an average size of approximately 5.39 mm in Orobelle and 4.8 mm in Bomby, as well as an average anther length of 2.75 mm in Orobelle and 2.71 mm in Bomby. Buds, calyx lengths and anther sizes were measured with the help of Magcam DC5 software under stereo microscope (Olympus SZX7, DF PLAPO1.25 X, Tokyo, Japan). Additionally, anthers without any purple pigmentation were selected in Orobelle, while in Bomby, anthers with slight to no pigmentation at the top of the anther sac end were chosen.

### Surface sterilization

The buds were gently washed with sterile distilled water to remove any external contaminants. Buds were first presterilized using 0.5% bavistin and few drops of tween 20 for 10 min under tap water and washing with autoclaved distilled water. Final surface sterilization was done in laminar air flow cabinet with 4% sodium hypochlorite for 10 min followed by three washings with autoclaved distilled water to remove the traces of sodium hypochlorite.

### In vitro Culture and Nutrient Media

The calyx and corolla of the buds were carefully dissected to expose the anthers while ensuring minimal damage to the reproductive tissues. Filaments were carefully removed from each anther without causing any injury to the anthers. Thereafter, anthers were cultured horizontally on culture medium. MS (Murashige and Skoog 1962), B5 medium (Gamborg et al. 1968) and Double layer medium (Supena et al. 2006) supplemented with different combinations and concentrations of growth regulators were used for the study. 2.6 g/L gelrite was used as the gelling agent in all the studies. Given the historically challenging nature of androgenesis in hot pepper cultivation, our research endeavors were directed towards optimizing or minimally modifying previously standardized media formulations (Dolcet – Sanjuan et al. 1997; Supena et al. 2006;Taskin et al. 2011; Ozsan and Onus,^ 2018^;) for androgenesis in various Capsicum genotypes across different parts of the world. The aim was to identify a genotype-independent medium suitable for inducing successful androgenesis in *Capsicum annuum* L. (Tables 1 and 2).

**Table 1:** Effect of different media types and combinations and concentrations of PGRs on direct regeneration from anther culture in Orobelle

Media code	Treatments	% Embryo formation (Mean ± S.E.)	% callus formation (Mean ± S.E.)	% Plant regenerated from embryo formed (Mean ± S.E.)	% Acclimatization (Mean ± S.E.)
M1	MS + 4 mg/L NAA + 1 mg/L BAP	1.85 ± 0.02^a^	13.80 ± 0.16^e^_2_	2.92 ± 0.03^a^	2.55 ± 0.06^a^
M2	MS + 4 mg/L NAA + 0.1 mg/L BAP	0.94 ± 0.02^b^	15.40 ± 0.15^d^_2_	2.12 ± 0.003^b^	1.58 ± 0.01^b^
M3	MS + 4 mg/L NAA + 0.5 mg/L BAP	0.84 ± 0.02^b^	17.84 ± 0.33^c^_2_	1.87 ± 0.02^c^	1.22 ± 0.003^c^
M4	MS + 5 mg/L NAA + 0.1 mg/L BAP + 0.05 mg/L biotin	0.59 ± 0.02^cd^	22.74 ± 0.58^a^_2_	0.71 ± 0.00^f^	0.71 ± 0.00^d^
M5	MS + 5 mg/L NAA + 1 mg/L BAP + 0.05 mg/L folic acid	0.49 ± 0.02^cd^	21.34 ± 0.16^b^_2_	0.71 ± 0.00^f^	0.71 ± 0.00^d^
M6	B5 + 4 mg/L NAA + 1 mg/L BAP	0.84 ± 0.02^b^	14.90 ± 0.01^d^_2_	1.58 ± 0.006^d^	1.22 ± 0.003^c^
M7	B5 + 4 mg/L NAA + 0.1 mg/L BAP	0.78 ± 0.02^b^	13.98 ± 0.04^e^_2_	1.58 ± 0.006^d^	1.22 ± 0.003^c^
M8	B5 + 4 mg/L NAA + 0.5 mg/L BAP	0.62 ± 0.02^c^	12.56 ± 0.22^f^_2_	1.22 ± 0.007^e^	0.71 ± 0.00^d^
M9	B5 + 4 mg/L NAA + 0.1 mg/L BAP + 1 g/L glutamine	0.51 ± 0.02^ cd^	15.36 ± 0.26^d^_2_	0.71 ± 0.00^f^	0.71 ± 0.00^d^
M10	B5 + 4 mg/L NAA + 1 mg/L BAP + 1 g/L glutamine	0.46 ± 0.02^de^	14.97 ± 0.03^d^_2_	1.23 ± 0.00^e^	0.71 ± 0.00^d^
M11	Double layer media—MS + 2%sucrose, 0.5% activated charcoal, gelrite- 2.6 g/L, Liquid upper layer contained half strength MS + 2% sucrose	0.37 ± 0.02^ef^	10.56 ± 0.03^g^_2_	0.71 ± 0.00^f^	0.71 ± 0.00^d^
M12	Double layer media—Nistch medium, Maltose- 2%, Activated Charcoal – 0.5%, gelrite- 2.6 g/L, Liquid top layer consists of Nistch medium and 2% maltose	0.32 ± 0.02^f^	9.50 ± 0.03^h^_2_	0.71 ± 0.00^f^	0.71 ± 0.00^d^
	CV	3.24	1.18	1.41	0.68

Media 1 to 10 – contain 0.25% activated charcoal, 15 mg/L AgNO3, 30 g/L sucrose and gelrite- 2.6 g/L in addition to the PGRs mentioned in the table

% Plant regeneration and % plant acclimatization is calculated based on the total number of embryo formed from each respective treatments

The mean data mentioned in the table are square root (% embryo formation, % callus formation % plant regenerated from embryo formed and % acclimatization) transformed values respectively

Values are mean ± standard error and treatment values followed by different letters are significant (α ≤ 0.05)

The culture medium was sterilized by autoclaving at 121 ° C @ 15 psi pressure for 20 min. pH of the medium was set to 5.8 by using 1N HCl and NaOH solution before autoclaving. In order to prevent denaturation of hormones, thermolabile hormones were first filter sterilized by using 0.20 micron syringe filters and then added to the warm (35–40 ° C) autoclaved media before solidifying. The cultures were incubated at 25 ± 2 °C under 16 h light and 8 h dark conditions from the beginning of the culture. The light intensity of 40 µmol m^−2^ s^−1^ was maintained with the help of cool white fluorescent lights

### Pre and post culture treatments

Buds were subjected to cold temperature pretreatment (CT1-CT6) at 4° C for 0–8 days and anthers were subjected to post-culture treatment at 32° C ( HT1-HT6) for 0–6 days. (Table 3).

### Effect of Season on embryo induction from anther cultures

The impact of seasonal variations on embryo induction from anther cultures was investigated by collecting flower buds from the target plant species throughout the year. The percentage of embryo formation on the medium showing best embryo induction was assessed based on cultured anthers obtained from buds collected during distinct periods: January-March, April-June, July–September, and October-December.

### Plant Regeneration from anther derived Microspore embryos

After the microspore embryos obtained from cultured anthers had germinated and attained a length of 0.3–0.5 cm, they were subcultured into test tubes containing regeneration medium (half strength MS medium, 2% sucrose, and 0.1 mg/L 6-benzylaminopurine (BA), solidified with 2.6 g/L gelrite and incubated with same growth conditions as mentioned above.

### Hardening and acclimation

Rooted plantlets were carefully removed with the help of forceps from the test tubes without damaging the root system and washed thoroughly using sterile distilled water to remove the traces of medium adhering to the root system. Thereafter, they were transferred into plastic pots of 10 cm size, half filled with pre-sterilised coco peat, vermiculite and perlite in the ratio of 3:1:1. Nutrients were supplied through the application of Hoagland solution at 3 days interval. Each pot was covered with polythene bag to maintain humidity around the plants and 2 to 3 holes were made in the polythene bags for proper aeration and these pots were kept in a culture room at a temperature of 25 ± 2° C and 16 h/8 h (light/dark) photoperiods.

The plants were exposed to outer environmental conditions for about 15–20 min during initial hardening days. Over the period of time, the time of exposure was increased gradually and finally after 25–30 days of hardening, the plants were ready for acclimatization under polyhouse condition. For acclimatization, the plants were transferred into pots containing autoclaved soil.

### Ploidy level analysis

To ascertain the ploidy status of the plant, two methods were employed. Firstly, a comparison was made between the phenotypic characteristics of plantlets derived from anther-derived microspores and diploid plants obtained through the in vitro culturing of seeds from the respective hybrids (Orobelle and Bomby). Secondly, flow cytometry was utilized to assess the ploidy level of the developed plantlets.

### Morphological Parameters

Forty days after hardening and acclimatization, anther culture derived plantlets as well as seed culture derived plantlets were compared for morphological traits such as plant height(cm), leaf length(cm), leaf width(cm) and internode length(cm) so as to have an idea of the vigour of plantlets derived obtained from anther culture derived microspores. Average of 8 plants were used to derive the data. Descriptive statistical analysis (mean and standard error) was used to interpret the data.

### Flow Cytometry

Ploidy level of anther derived plantlets was analysed using flow cytometry (Sysmex Partec). For flow cytometry, green young leaves from microspore derived plantlets were taken and checked against the diploid control as mentioned below.

To set the control, seeds of the diploid plant taken as control were crushed in a small volume of Cystein UV ploidy staining solution. Additional staining solution was added to prepare a homogenous solution and then it was passed through a 30 µm filter by tapping. Before reading the control, the sample insertion tube was washed with milliQ water. The sample was thereafter placed in sample insertion tube and reading was taken. The gain and threshold of peak was adjusted and the peak for diploid control was saved. The readings of the test samples (from plantlets derived from microspores) were then taken following a similar procedure. One cm leaf disc from the topmost young leaf of the target plantlets were used for the purpose. Five morphologically healthy plantlets of each genotype were tested using flow cytometry.

### Data collection and statistical analysis

The experiment was laid out in a completely randomized design which included three replications for the treatments. Each replication included forty petriplates each with 30–35 anthers. One way ANOVA was performed for the data collected for each of the studied variable. After completing analysis of variance, Post hoc study was performed using Tukey’s method for the pairwise treatment comparison and ranking. SAS software was used for the statistical analysis. Square root transformation was used when the data ranged between 0–30% or 70–100% (Bartlett 1947).

## Results

Under the present study, the effect of pre and post culture treatments, different nutrient media, growth hormone concentration, season as well as regeneration efficiency of two pepper hybrids on haploid induction and production was investigated.

### Selection of flower buds

**Fig. 1:**
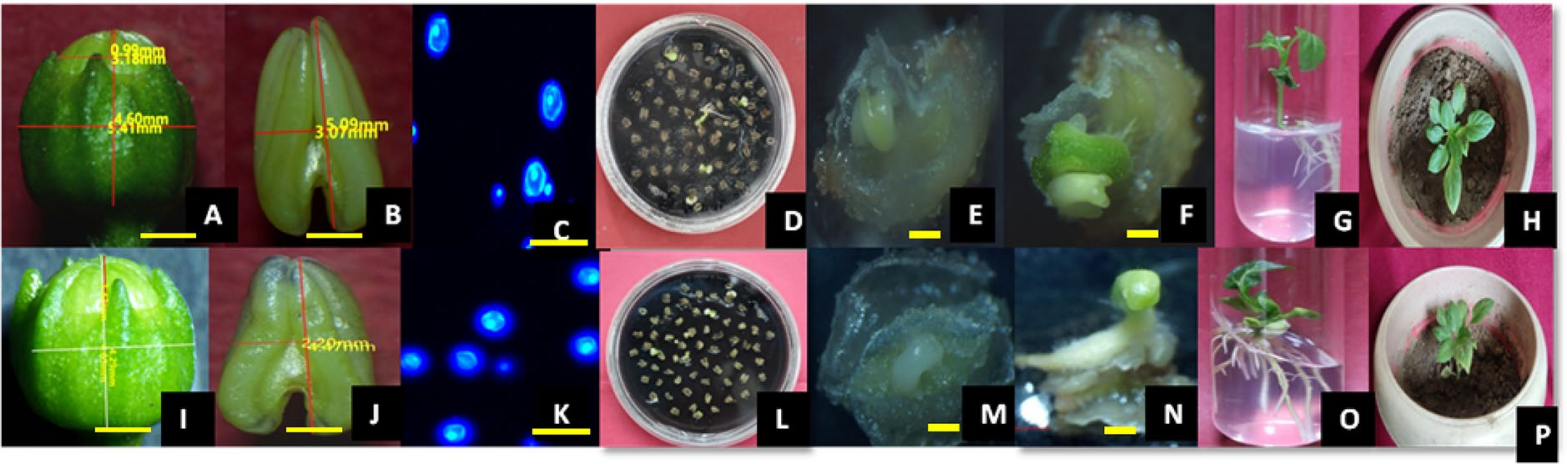
Stages of microspore embryogenesis in Orobelle (A-H) and Bomby (I-P). A-C, I-K: Represents flower bud, anther (Scale Bar: 1 mm) and microspore (Scale Bar: 10 µm)at uninucleate stageD-E, L-M: Embryo formation after 6 & 7 weeks of culture(Scale Bar: 1 mm), F-G, N–O: Embryo formation after 8 & 9 weeks of culture and H, P: hardened plant

Anthers were carefully collected from unopened flower buds, measuring approximately 5.39 mm in Orobelle and 4.8 mm in Bomby. These buds contained uninucleate microspores with a notable large cytoplasmic cavity and the nucleus positioned towards the exine. This particular developmental stage is known to greatly contribute to successful androgenesis in pepper breeding. In line with our previous research (Sahana et al. 2021), the selected buds had either equal lengths of corolla and calyx or a slightly longer corolla. Additionally, Orobelle anthers lacked purple pigmentation, while Bomby anthers showed slight to no pigmentation at the top of the sac as depicted in Fig. 1 (A-C, I-K).

### Influence of different types and components of culture media on microspore directed embryogneneis in pepper anther culture

Tables 1 and 2 present data showcasing the impact of various media types and concentrations of plant growth regulators on anther derived microspore embryo induction in the two test genotypes. Notably, MS medium exhibited superior performance when compared to B5 medium. Among the different combinations of growth regulators, the best results were achieved using M1 medium ie MS medium supplemented with 4 mg/L NAA, 1 mg/L BAP, 0.25% activated charcoal, 15 mg/L AgNO3, and 30 g/L sucrose, which resulted in the highest percentage of embryo formation (1.85% in Orobelle and 1.46% in Bomby). In the case of Bomby, the second-best medium was found to be M2 (MS + 4 mg/L NAA + 0.1 mg/L BAP). Similarly, for Orobelle, M2, M3 (MS + 4 mg/L NAA + 0.5 mg/L BAP), M6 (B5 + 4 mg/L NAA + 1 mg/L BAP), and M 7 (B5 + 4 mg/L NAA + 0.1 mg/L BAP) exhibited superior values of percent embryo formation compared to other media (0.94%, 0.84%, 0.84%, and 0.78% respectively).

**Table 2:** Effect of different media types and combinations and concentrations of PGRs on direct regeneration from anther culture in Bomby

Media code	Treatments	% Embryo formation (Mean ± S.E.)	% callus formation (Mean ± S.E.)	% Plant regenerated from embryo formed	% Acclimatization
M1	MS + 4 mg/L NAA + 1 mg/L BAP	1.46 ± 0.02^a^	14.34 ± 0.24^e^_2_	2.35 ± 0.003^a^	1.87 ± 0.003^a^
M2	MS + 4 mg/L NAA + 0.1 mg/L BAP	0.83 ± 0.02^b^	14.37 ± 0.28^e^_2_	1.87 ± 0.018^b^	1.22 ± 0.003^b^
M3	MS + 4 mg/L NAA + 0.5 mg/L BAP	0.64 ± 0.02^c^	17.94 ± 0.11^c^_2_	1.58 ± 0.006^c^	1.22 ± 0.003^b^
M4	MS + 5 mg/L NAA + 0.1 mg/L BAP + 0.05 mg/L biotin	0.58 ± 0.02^ cd^	23.55 ± 0.16^a^_2_	1.22 ± 0.009^d^	0.71 ± 0.00^c^
M5	MS + 5 mg/L NAA + 1 mg/L BAP + 0.05 mg/L folic acid	0.41 ± 0.02^ef^	22.64 ± 0.48^b^_2_	0.71 ± 0.00^e^	0.71 ± 0.00^c^
M6	B5 + 4 mg/L NAA + 1 mg/L BAP	0.73 ± 0.02^bc^	15.85 ± 0.07^d^_2_	1.22 ± 0.007^d^	0.71 ± 0.00^c^
M7	B5 + 4 mg/L NAA + 0.1 mg/L BAP	0.70 ± 0.02^bc^	14.23 ± 0.17^e^_2_	1.87 ± 0.015^b^	1.22 ± 0.009^b^
M8	B5 + 4 mg/L NAA + 0.5 mg/L BAP	0.60 ± 0.02^ cd^	13.00 ± 0.14^f^_2_	0.71 ± 0.00^e^	0.71 ± 0.00^c^
M9	B5 + 4 mg/L NAA + 0.1 mg/L BAP + 1 g/L glutamine	0.49 ± 0.02^de^	16.55 ± 0.29^d^_2_	0.71 ± 0.00^e^	0.71 ± 0.00^c^
M10	B5 + 4 mg/L NAA + 1 mg/L BAP + 1 g/L glutamine	0.41 ± 0.02^ef^	14.24 ± 0.33^e^_2_	0.71 ± 0.00^e^	0.71 ± 0.00^c^
M11	Double layer media—MS + 2%sucrose, 0.5% activated charcoal, gelrite- 2.6 g/L, Liquid upper layer contained half strength MS + 2% sucrose	0.31 ± 0.02^ fg^	11.29 ± 0.26^g^_2_	0.71 ± 0.00^e^	0.71 ± 0.00^c^
m12	Double layer media—Nistch medium, Maltose- 2%, Activated Charcoal – 0.5%, gelrite- 2.6 g/L, Liquid top layer consists of Nistch medium and 2% maltose	0.26 ± 0.02^ g^	10.67 ± 0.25^g^_2_	0.71 ± 0.00^e^	0.71 ± 0.00^c^
	CV	3.56	2.82	1.11	0.56

Media 1 to 10 – contain 0.25% activated charcoal, 15 mg/L AgNO3, 30 g/L sucrose and gelrite- 2.6 g/L in addition to the PGRs mentioned in the table

% Plant regeneration and % plant acclimatization is calculated based on the total number of embryo formed from each respective treatments

The mean data mentioned in the table are square root (% embryo formation, % callus formation % plant regenerated from embryo formed and % acclimatization) transformed values respectively

Values are mean ± standard error and treatment values followed by different letters are differ significantly (α ≤ 0.05)

MS medium (M4) supplemented with 5 mg/L NAA, 0.1 mg/L BAP, 0.25% activated charcoal, 15 mg/L AgNO3, and 30 g/L sucrose exhibited the highest percentage of callus formation (22.74% in Orobelle and 23.55% in Bomby). In contrast, the two double layer media employed in the study yielded inferior results compared to other media, both in terms of microspore embryo induction from anthers and percent callus formation in both genotypes (Tables 1 and 2).

After 25–50 days of culture (depending on the type of media), responsive anthers swelled and increased in size, while the others remained same, became dark and lost their turgescence. Following this, after 35–60 days (depending on the type of media), responsive anthers opened along the dehiscence line, from where the microspore directed embryo formation occurred. The direct emergence of embryos from microspores, as opposed to other parts of anthers, represents a vital consideration when choosing the induction medium for capsicum. This fundamental criterion ensures the purity of haploid plantlets, completely devoid of any genetic contributions from non-microspore sources within the anthers. In M1 medium, the induction of embryos was achieved within a relatively short timeframe of 60–70 days, while in M2 medium the induction of embryos occurred after a longer duration of 110 days. Conversely, when utilizing B5 medium, the duration for embryo induction ranged from 85 to 110 days.

### Proliferation of microspore embryos derived from anther culture and regeneration of Plantlets

**Fig. 2:**
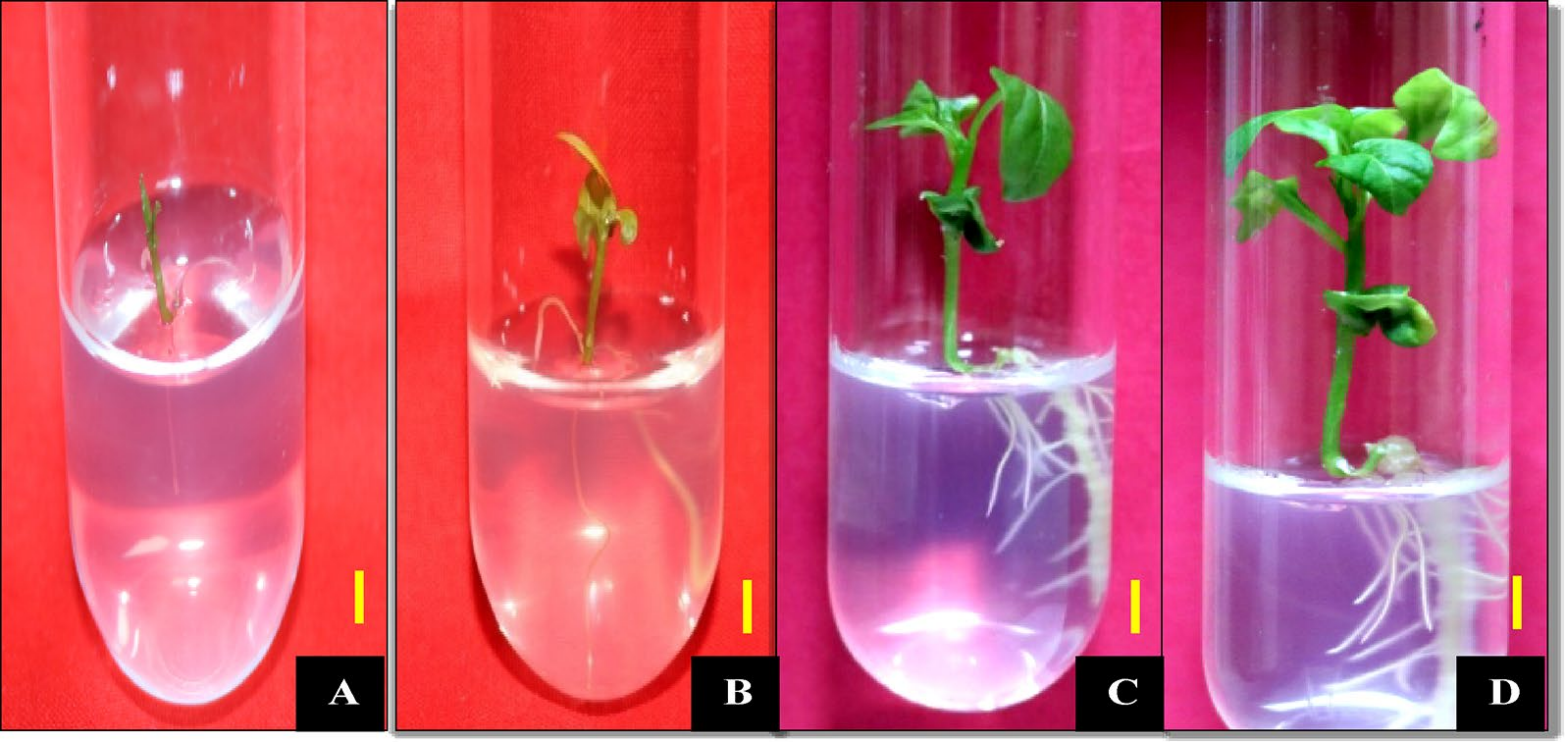
Anther derived plantlet at different days after proliferation. A-D: 30, 40, 50 and 60 days after proliferation respectively (Scale Bar: 1 cm)

The embryoids obtained were carefully transferred to half strength MS medium with 2% sucrose, supplemented with 0.1 mg/L 6-benzylaminopurine (BA), solidified using 2.6 g/L gelrite. These cultures were maintained under optimal conditions of 16 h of light and a temperature of 25 °C. Initially, the growth rate was notably sluggish, with only few plantlets achieving an average length of approximately 3 cm after a period of 40 days upon transfer to the regeneration medium and many embryos did not show further growth after transfer. In addition some of the embryos resulted in only rootlike structures while some embryos formed only shoots, these abnormal plantlets did not show much growth later and eventually died. In the remaining embryos, a gradual and steady progression was witnessed in shoot length, root growth, leaf count, and stem girth (Fig. 2). After a total culture duration of 60 days, the plantlets obtained from healthy embryos exhibited shoot length of 6–7 cm, root length of 5–6 cm, and a leaf count ranging from 6 to 8 leaves. The plants were hardened and acclimatized as mentioned in material and methods section. In total, we obtained 486 and 490 anther-derived embryos in Orobelle and Bomby, respectively, from all the treatments tested. Out of these, 12 and 11 plantlets successfully developed a complete root and shoot system in Orobelle and Bomby, respectively. The remaining embryos resulted in plantlets with abnormalities such as improper root and shoot development or no further growth. Ultimately, 10 and 8 plants were obtained after hardening in Orobelle and Bomby, respectively.

### Effect of pre and post culture treatment

Table 3 illustrates the impact of cold pretreatment on the percentage of embryo formation in both genotypes. It was found that subjecting the buds to a cold pretreatment at 4 °C for varying durations had a significant influence. Notably, the highest rate of embryo induction was achieved with a pretreatment duration of 4 days (CT4), resulting in a 3.05% and 2.72% embryo formation in Orobelle and Bomby, respectively. However, it is worth noting that post-culture incubation of anthers at 32 °C led to browning of cultured anthers, even after just one day of incubation.

**Table 3:** Effect of pre/post culture treatment on embryo formation in Orobelle and Bomby

Sl. No	Treatments	Pre culture treatment at 4 °C (in days)	Percent embryo formation		Treatments	Post culture treatment at 32 °C(in days)	Remarks
			**Orobelle** **(Mean ± S.E.)**	**Bomby** **(Mean ± S.E.)**			
1	CT1	0	1.94 ± 0.19^d^	1.75 ± 0.18^d^	HT1	0	Anthers turned brown in all the treatments
2	CT2	1	2.27 ± 0.19^c^	1.98 ± 0.18^c^	HT2	2	
3	CT3	2	2.36 ± 0.19^b^	2.12 ± 0.18^b^	HT3	3	
4	CT4	4	3.05 ± 0.19^a^	2.72 ± 0.18^a^	HT4	4	
5	CT5	6	1.87 ± 0.19^e^	1.63 ± 0.18^e^	HT5	5	
6	CT6	8	1.75 ± 0.19^f^	1.46 ± 0.18^f^	HT6	6	
			21.48	23.07			

The mean data mentioned in the table are square root transformed values

Values are mean ± standard error and treatment values followed by different letters are significant (α ≤ 0.05)

### Effect of Season on embryo induction

**Fig. 3:**
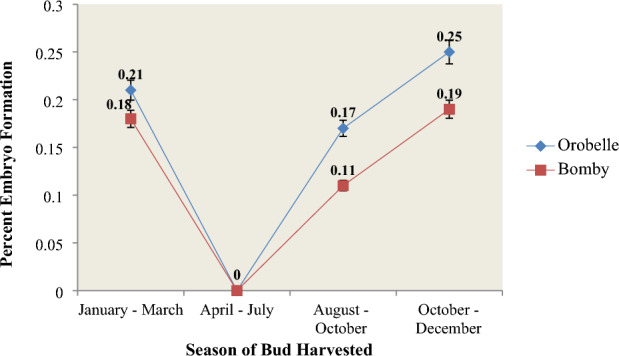
Effect of season on regeneration potential of Orobelle and Bomby

Anthers were cultured on the MS medium supplemented with 4 mg/L NAA, 1 mg/L BAP, 0.25% activated charcoal, 15 mg/L AgNO3, and 30 g/L sucrose throughout the year. Anther-derived microspores showed best germination (0.25% in Orobelle and 0.19% in Bombay) when anthers were collected from the polyhouse in October-December (autumn). Successful plant regeneration with good root and shoot systems was achieved during the winter. No embryos were formed from anthers cultured in either genotype during April-July. It was observed that during the months April-July, due to high temperature conditions, buds in both the pepper genotypes were smaller as compared to their respective bud sizes in low temperature conditions. Also, in low temperature conditions, the anthers of both the pepper genotypes were observed to have pale and brownish yellow appearance.These findings suggest that plants grown under low temperature conditions are more responsive to androgenesis (Fig. 3).

### Plant morphological parameters

**Fig. 4:**
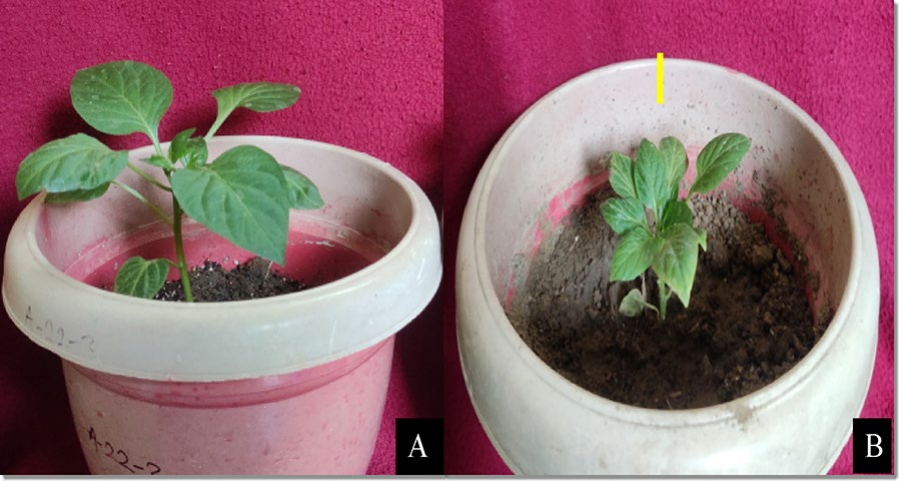
A: In vitro raised diploid plant and B: Anther culture derived plant (Scale Bar: 5 cm)

**Fig. 5:**
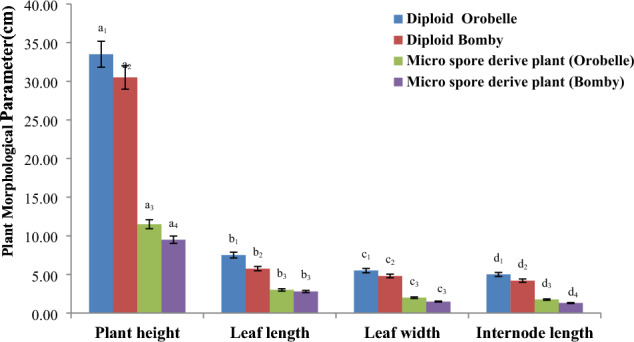
Plant morphological characters of diploid and anther derived plant of Orobelle and Bomby. *Different letters for a given character (a,b,c,d) and different number indicate that  there is a significant difference with respect to plant height, leaf length, leaf width and internode length in the test plants of different origin

Morphological variations could be discerned between diploid and anther derived plants of both genotypes following a 40-day acclimatization period (Figs. 4 and 5). These variations indicated a discernible decline in the vigour of anther derived plants when compared to their seed derived counterparts. The anther-derived plants were significantly smaller and less vigorous than the seed-derived plants. This was likely due to the fact that the anther-derived microspore plants had haploid sets of chromosomes, while the seed-derived plants had diploid sets.

The average plant height of anther-derived plants was 15.50 cm and 18.5 cm in Bomby and Orobelle, respectively. The average leaf length was 3.79 cm and 5.5 cm, the average leaf width was 2.29 cm and 2.89 cm, and the average internode length was 2.45 cm and 2.95 cm in Bomby and Orobelle, respectively. The corresponding values in seed-derived diploid plants of Bomby and Orobelle respectively were 40.5 cm and 43.5 cm for plant height, 11.5 cm and 16.5 cm for leaf length, 5.79 cm and 6.49 cm for leaf width, and 6.2 cm and 7.8 cm for internode length. These findings suggest that anther-derived microspore plants had reduced vigor compared to seed-derived plants, likely due to the haploid nature of the anther-derived microspore plants.

### Flow cytometry

**Fig. 6:**
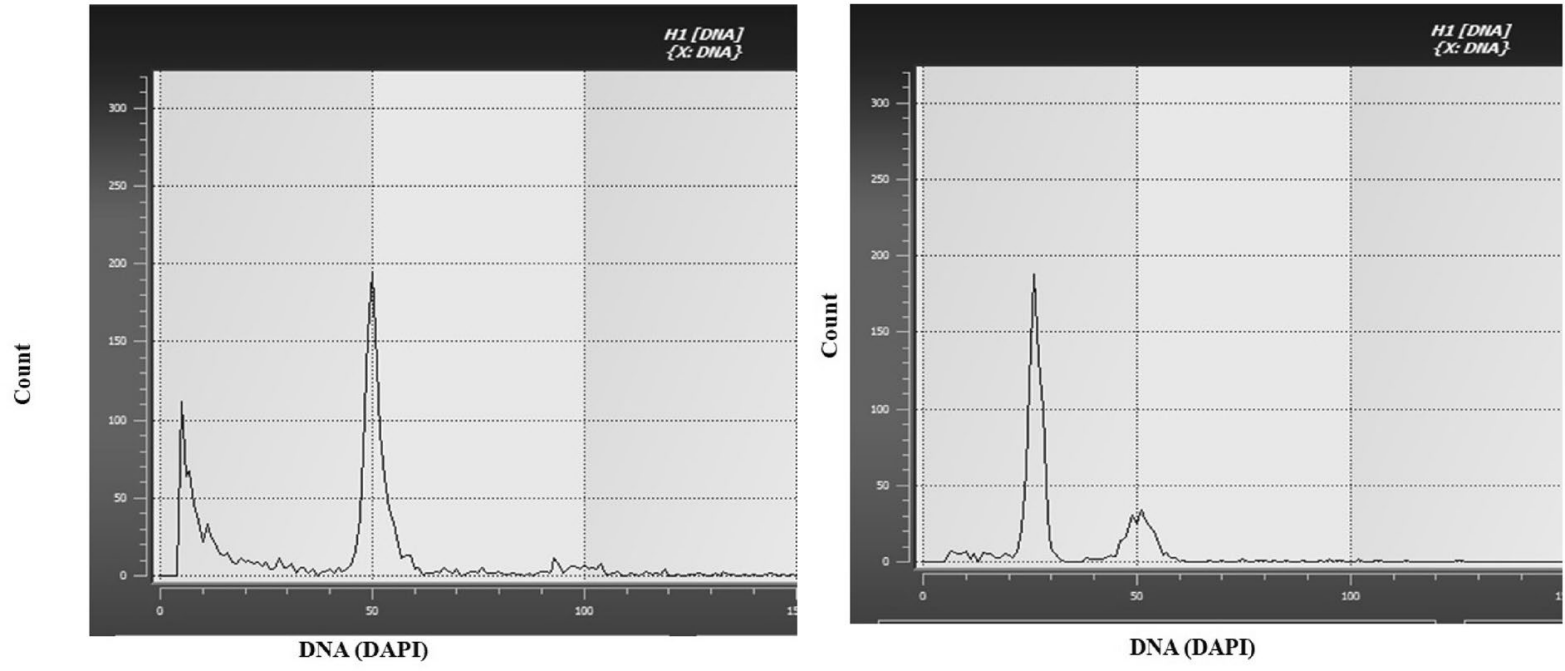
Ploidy estimation of microspore derived plantlets with G1 DNA peak set at channel number 25 (B) against a Diploid control with G1 DNA peak set at channel 50 (A)

For measuring the chromosomal status of seeds derived diploid plants peak was adjusted at a gain of 50. When the observation were taken from the microspore derived plantlets gain was observed at 25 confirming the haploid nature of microspore derived plantlets. (Fig. 6).

## Discussion

Conventional methods of plant breeding are characterized by extensive time requirements encompassing screening, line development, evaluation, and crossing to attain homozygous lines. However, biotechnological applications present an array of powerful tools, especially in the realm of plant breeding and tissue culture. Notably, the utilization of haploid and doubled haploid technology proves to be an effective means of selecting superior plants, while significantly reducing the time needed for generational advancements.

Through doubled haploidization, it becomes possible to develop homozygous plants within a single generation, thus presenting an opportunity to introduce novel varieties in self-pollinated crops or to establish parental inbred lines for hybrid production in cross-pollinated crops. These homozygous doubled haploid plants serve as a foundational platform for precise phenotyping of various traits, genetic studies, QTL mapping, and transcriptomics investigations (Dwivedi et al. 2015). Moreover, haploidy can serve as a selection tool for eliminating genotypes that exhibit notable inbreeding depression in cross-pollinated crops. Doubled haploid lines also hold immense value in the fields of marker-assisted selection (MAS) and functional genomics, further augmenting their significance (Ren et al.^2017^).

Pepper, standing as the third solanaceous crop in line after tomato and eggplant, has been recognized for its recalcitrance when it comes to androgenesis induction (S﻿egui Simarro et al. 2011). Notably, the success of anther culture has been documented to rely on various factors, including genotype (Kim et al. 2004; Supena et al. 2006; Ari et al. 2016a, b), developmental stage of flower buds (ParraVega et al. 2013; Mangal and Srivasatava 2019), nutrient medium (Supena et al. 2006; Irikova et al.2011a, b; Bat et al. 2020), anther pretreatments (Dumas de Vaulx et al. 1981), and even the season of culture (Ercan et al.^2006^; Rodeva and Cholakov, 2006; Taskin et al. 2011). In our previous investigations, the microspore/pollen stage within the anthers was precisely determined through the implementation of the DAPI (DAPI-4′,6-diamidino2-phenylindole) staining method (Mangal and Srivasatava 2019; Sahana et al. 2021).

The present study sheds light on the fact that direct regeneration of plants within both tested genotypes was successfully induced on MS medium enriched with 4 mg/L NAA, 1 mg/L BAP, 0.25% activated charcoal, 2.6 g/L gelrite, 30 g/L sucrose, and 15 mg/L silver nitrate. This outcome underscores the essentiality of a combined auxin and cytokinin regimen for successful embryo and plant regeneration in bell pepper, as previously reported by Ata et al. (2018), Bat et al. (2020), and Supena et al. (2006). Furthermore, the germinated embryos were subsequently transferred to half-strength MS medium supplemented with 0.1 mg/L BAP to facilitate the regeneration of plantlets. These growth regulator concentrations and combinations align with those employed by Supena et al. (2006), yielding favorable outcomes in the present experiment. In the present study, we tested the addition of biotin or folic acid at a concentration of 0.05 mg/L to the MS medium, as well as glutamine at a concentration of 1 mg/L to the B5 medium as reported by Ozsan and Onus (2018). However, our results were not consistent with those as these additions did not lead to any improvement in the induction rate. The variation in outcomes could potentially be attributed to differences in genotypic responsiveness or the specific experimental conditions employed, which may influence the effectiveness of these supplementation strategies. Further investigation is required to elucidate the underlying factors contributing to this discrepancy.

Stress treatments encompassing extreme temperatures, osmotic variations, and physiological stress play a pivotal role in the transition of microspores from the gametophytic to the sporophytic embryo stage. In the present study, the pre-culture cold treatment of flower buds at 4 °C for 4 days, yielded early and normal looking embryo formation in both genotypes. These findings align with the observations of Supena et al. (2006), Luitel and Kang (2013), Popova et al. (2016) and Solin et al. (2020) who also noted a positive impact on embryo formation and the emergence of normal-looking embryos when buds were pre-treated at 4 °C. However, the statistical significance of these effects was not consistently observed due to relatively high standard errors induced by the inherent heterogeneity within the donor plant populations.

In contrast, the post-culture treatments of anthers at 32 °C, as explored in the present investigation, proved ineffective as the anthers exhibited a browning phenomenon across all attempted durations. These results mirror the observations of Luitel and Kang (2013), who reported a negative effect on embryo generation with prolonged exposure to high temperatures (35 °C), indicating a decline in embryo production with longer durations. Notably, the shortest period of high-temperature treatment of 4 days has been reported to be the most effective by Veronica et al. (2013).

The success of anther culture is heavily influenced by the growing conditions and physiological state of the donor plants, which directly impact the quantity of pollen grains, endogenous hormone levels, and nutritional status of anther tissues, subsequently influencing the in vitro induction of embryogenesis in pepper (Irikova et al.2011a, b). Our findings unveiled that the optimal embryo induction rates (0.25 and 0.19% respectivey in Orobelle and Bomby) were achieved when microspores derived from anther culture were initiated during the autumn season (October to December). Furthermore, successful plant regeneration accompanied by robust root and shoot systems was consistently achieved during the winter season. These observations align with the suggestions of Ari et al. (2016a, b, c) and R'him et al. (2010), who also indicated that the autumn season yields promising embryo formation in capsicum genotypes when utilizing the shed-microspore culture technique. However, Ellialtıoğluet al. (2001) discovered that donor plants cultivated during the normal growing season of various species exhibited higher haploid performance compared to those grown outside of the typical growth season. Additionally, Olszewska et al.(2011) proposed that the reduced efficacy of androgenesis in the capsicum genus, which originates from tropical and subtropical climate zones, may be attributed to unpredictable temperature fluctuations during donor plant growth.

Phenotypic variations among regenerated plants from anther culture have been reported, encompassing differences in chlorophyll content (albinism) and morphological traits such as habit, leaves, flowers, inflorescences, or fruits (Kozik et al. 2002; Zagorska et al. 2004; Guo et al. 2005). In addition microspore-derived embryos have been observed to exhibit varied anomalies within the shoot apical meristem and its resultant organs, encompassing issues such as reduced cotyledon size to the complete disorganization of shoot apices. These abnormalities had a consequential impact on the germination process in numerous instances. (Kozik et al. 2002; Zagorska et al. 2004; Guo et al. 2005; Salas et al. 2011). Initial determinations, including the positioning of initials, can be influenced by callus pressure, impacting developing embryos in their early stages (Benelli et al. 2010). Genotype-dependent responses to varying culture conditions contribute to abnormalities in somatic embryo development, likely induced by inadequate culture conditions. Insufficient conversion or low conversion rates of somatic embryos are primarily linked to abnormalities in the shoot apical meristem or abnormal protoderm formation, leading to developmental arrest. Albinism, characterized by well-structured chlorophyll-deficient embryos has also been observed, and the frequency of albino plants may be associated with the age of anthers (Huang 1986). Many structures exhibit inherent imperfections during initial growth, such as slender stems and individual leaves, while others suggest deviations from normal ontogenesis at later stages. These variations may stem from recessive mutations that become apparent in haploid or doubled haploid regenerated plants. In some instances, changes in chromosome number and structure, cytoplasmic alterations, or mutations induced during the process may contribute to such variations. The frequency of genetic variations tends to be higher when there is an intervening callus phase in the in vitro plant regeneration process from anther cultures (indirect androgenesis). This intermediary phase increases the likelihood of diploid embryo formation, which could arise from anther walls. Notably, in the present work, no callus formation was observed prior to plant regeneration from microspore embryos, resulting in the absence of obvious phenotypic variations among anther-regenerated plants.

Determining the ploidy status of anther-derived plants is crucial to ensure their direct derivation from microspores. Haploid pepper plants are often characterized by distinct morphological features such as shorter plant height, narrower leaves, and shorter internodes, exhibiting reduced growth compared to diploid plants (Grozeva et al. 2021). Consistent with these observations, the microspore-derived plants in our study exhibited significantly reduced vigor compared to seed-derived plants, indicating their haploid nature. This was further confirmed through ploidy analysis using flow cytometry.

## Conclusion

In conclusion, this research highlights the significant advantages of in vitro anther-derived microspore culture as a rapid and efficient method for generating homozygous lines in pepper breeding programs. Our research findings indicate that the highest observed rates of embryo formation were 1.85% in the Orobelle genotype and 1.46% in the Bomby genotype. These results further support the prevailing notion of the recalcitrant nature of Capsicum species in terms of androgenesis. The limited success in achieving higher rates of embryo induction highlights the need for continued efforts to overcome the inherent challenges associated with androgenesis in Capsicum, thereby facilitating advancements in crop improvement and breeding strategies for this important crop. Our results indicate that the success of anther culture can be attributed to three key factors: (1) cultivating donor plants during the autumn season (October to December), (2) subjecting buds to cold pretreatment at 4 °C for 4 days, and 3) conducting anther culture in MS medium fortified with 4 mg/L NAA, 1 mg/L BAP, 0.25% activated charcoal, 2.6 g/L gelrite, 30 g/L sucrose, and 15 mg/L silver nitrate. These findings underscore the immense potential of anther culture and microspore embryogenesis as a revolutionary technique in accelerating pepper breeding programs. By enabling the rapid production of superior homozygous lines within a single generation, this method offers a promising avenue for enhancing the efficiency and speed of pepper cultivar development. It provides breeders with a powerful tool to expedite the selection of desirable traits, streamline the evaluation process, and ultimately contribute to the development of improved pepper varieties.

## Data Availability

The datasets used and/or analysed during the current study are available from the corresponding author on reasonable request.

## Abbreviations

MS: Murashige and Skoog
B5: Gamborg
BAP: Benzyl amino purine
NAA: α–Naphthalene acetic acid
AgNO3: Silver nitrate
HCl: Hydrochloric acid
NaOH: Sodium hydroxide
DAPI: 4′,6-Diamidino2-phenylindole
QTL: Quantitative trait loci
MAS: Marker-assisted selection
ANOVA: Analysis of variance
SE: Standard Error
CV: Coefficient of variation
